# Peritoneal Malignant Psammomatous Mesothelioma

**DOI:** 10.4021/wjon230w

**Published:** 2010-08-29

**Authors:** Teresa Pusiol, Maria Grazia Zorzi, Doriana Morichetti, Irene Piscioli, Michele Scialpi

**Affiliations:** a Institute of Anatomic Pathology, S.Maria del Carmine Hospital, Rovereto – Rovereto – Trento, Italy; b Department of Radiology, Budrio Hospital, via Benni 44, 40054 Budrio (BO), Italy; c Department of Surgical, Radiologic and Odontostomatologic Sciences, Section of Diagnostic and Interventional Radiology, University of Perugia, S. Maria della Misericordia Hospital, S. Andrea delle Fratte, 06156 Perugia, Italy

**Keywords:** Psammoma bodies, Malignant mesothelioma, Psammomatous malignant mesothelioma

## Abstract

Psammoma bodies (PBs) are observed most commonly in papillary thyroid carcinoma, meningioma, and papillary serous cystadenocarcinoma of the ovary. We report one case of peritoneal malignant mesothelioma (PMM) with massive deposition of PBs. A 72-years-old man presented with abdominal swelling and marked weight loss. Contrast enhanced computed tomography showed fluid diffuse in peritoneal recesses, thick septa with micronodules in the greater omentum and adjacent enhancement of the thickened peritoneum. The explorative laparoscopy showed diffuse minute parietal peritoneal nodules. The peritoneal biopsy revealed a superficial papillary growth of malignant epithelial-like cells with diffuse involvement of submesothelial tissues. Massive deposition of PBs was observed. Nuclear and cytoplasmic calretinin immunoreactivity was present in neoplastic cells along with membranous D2-40 and membranous/cytoplasmic cytokeratin 5 staining. The patient was treated with chemotherapy (gemcitabine, vinorelbine, cisplatin). PBs may represent an active biologic process ultimately leading to degeneration/death of tumor cells and retardation of growth of the neoplasm. It may also serve as a barrier against the spread of tumor. Psammomatous malignant mesothelioma may simulate serous psammocarcinoma of the peritoneum. The behavior of serous psammocarcinoma is more closely similar to borderline serous tumor than to serous carcinoma. Further studies are necessary to establish if massive deposition of PBs may define a new variant of psammomatous malignant mesothelioma with a favorable impact to the prognosis of usual psammomatous malignant mesothelioma, as well as in serous psammocarcinoma of the peritoneum.

## Introduction

Psammoma bodies (PBs) are concentrically laminated calcific spherules that occasionally appear cracked (psammos [“sand”] + oma [“tumor”]). PBs are observed most commonly in papillary thyroid carcinoma, meningioma, and papillary serous cystadenocarcinoma of the ovary [1]. PBs have been reported rarely in other neoplasms and benign non-neoplastic conditions. We report one case of peritoneal malignant mesothelioma (PMM) with massive deposition of PBs with emphasis to biological significance of PBs and diagnostic differentiation with similar neoplasms.

## Case Report

**Figure 1:**
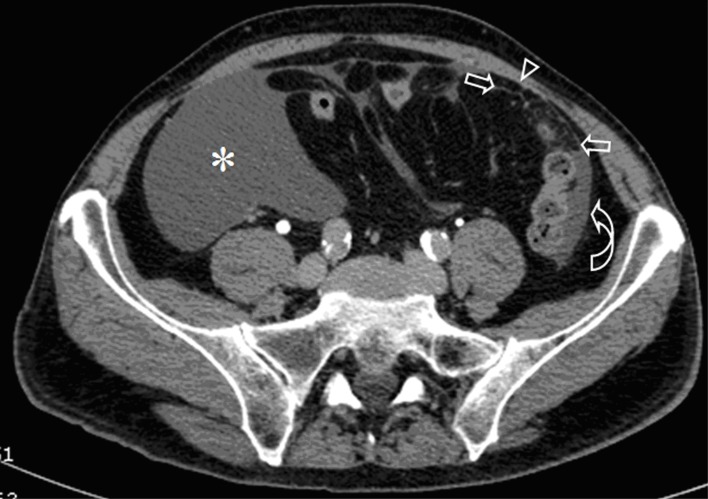
Contrast-enhanced CT of the abdomen shows abundant peritoneal fluid in the right iliac fossa (*) and paracolic fluid in the left side (curved arrow). Note the thick septa with micronodules in the greater omentum (arrows) and circumscribed enhancement of the thickened peritoneum (arrow head).

**Figure 2:**
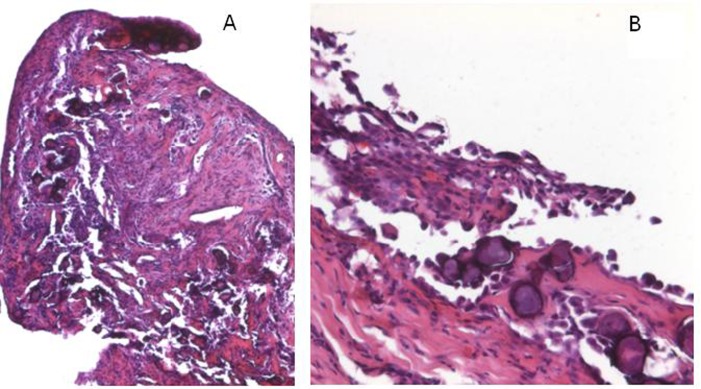
A. The peritoneal bioptic fragment shows massive deposition of Psammoma bodies (H&E; 25X). B. The superficial peritoneum shows a proliferation of malignant cells with massive presence of psammoma bodies (H&E; 100X).

**Figure 3:**
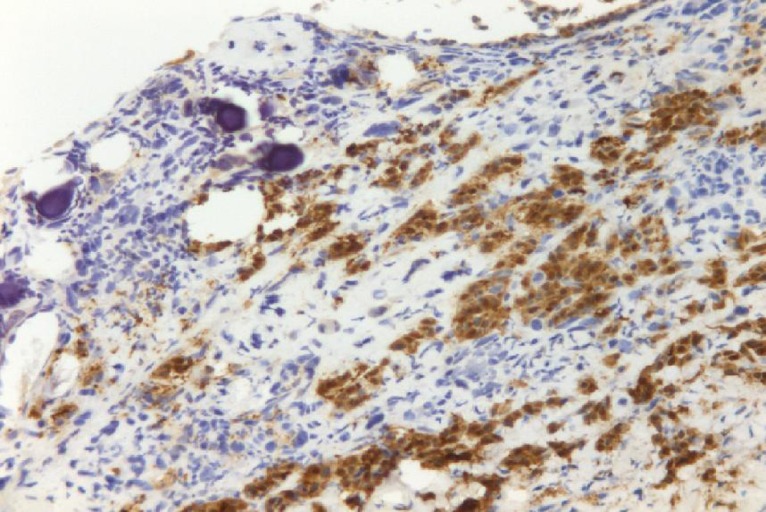
Neoplastic cells show nuclear and cytoplasmic calretinin immunoreactivity.

**Figure 4:**
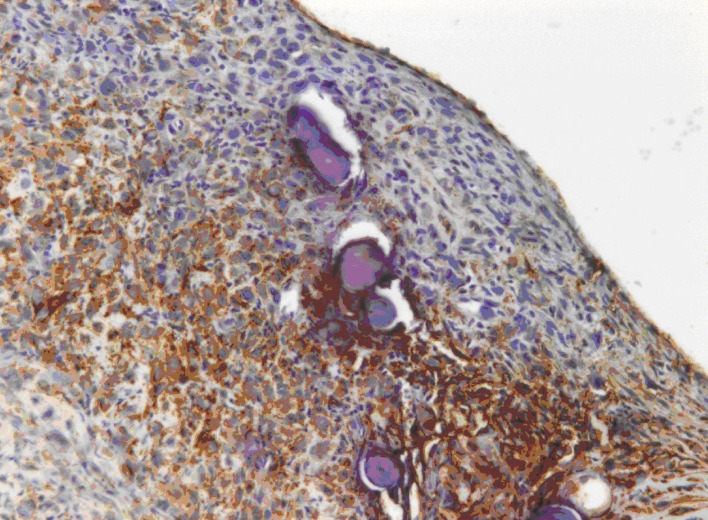
A positive membranous staining with the D2-40 antibody is seen in neoplastic cells.

A 72-year-old man presented with abdominal swelling and marked weight loss. No history of exposure to asbestos was found. Abdominal ultrasound revealed abundant perihepatic, perisplenic and pelvic fluid. Contrast enhanced computed tomography showed fluid diffuse in peritoneal recesses, thick septa with micronodules in the greater omentum and adjacent enhancement of the thickened peritoneum (Fig. 1). Malignant cells were found in the cytologic analysis of ascites. The explorative laparoscopy showed diffuse minute parietal peritoneal nodules. The peritoneal biopsy revealed a superficial papillary growth of malignant epithelial-like cells with diffuse involvement of submesothelial tissues. Massive deposition of PBs was observed (Fig. 2 A and B). Nuclear and cytoplasmic calretinin immunoreactivity was present in neoplastic cells (Fig. 3), along with membranous D2-40 (Fig. 4) and membranous/cytoplasmic cytokeratin 5 staining. The patient was treated with chemotherapy (gemcitabine, vinorelbine, cisplatin).

## Discussion

PMM is universally regarded as a fatal disease (median survival 6-12 months; mean symptoms-to survival time 345 days [2-3]. In the recent World Health Organization Histological Typing of Lung and Pleural Tumors, diffuse malignant mesotheliomas have been divided into epithelioid, sarcomatoid, desmoplastic and biphasic [4]. PMMs are qualitatively similar to those occurring in the pleural cavity, but the relative proportions among the various types and the criteria used for differential diagnosis with metastatic carcinoma (ovary and lung respectively) are different. Other variants of PMM are MM with deciduoid features and lymphohistiocytoid MM [5, 6]. A precise diagnosis based on imaging findings alone is not possible. Furthermore, distinguishing a benign from a malignant process as well as a primary from a metastatic process is also challenging. Therefore, the definitive diagnosis of PMM depends on histological and immunohistochemical examination. The frequency of PBs presence in PMMs is undetermined but certainly their presence is a very unusual finding.

Attanoos and Gibbs reported that PBs may be seen in approximately 5% to 10% cases of PMMs [7]. But these authors have not documented this finding with a series of cases or with citations reporting this value. In well-differentiated papillary mesothelioma of peritoneum, PBs have been described in 22% of the cases [8]. In the first report of this indolent, benign disease, the illustration of PBs showed only two psammoma bodies and documented that the presence of PBs is very limited. To date massive depositions of PBs have not been reported in PMM. The pathogenesis of extensive presence of PBs is unknown. According to Das et al [9] we believe that single necrotic cells constitute seed crystals that become incrusted with the mineral deposits and the progressive acquisition of outer layers may create its lamellated configurations. PBs may represent an active biologic process ultimately leading to degeneration/death of tumor cells and retardation of growth of the neoplasm. It may also serve as a barrier against the spread of tumor. Psammomatous MPM may simulate serous psammocarcinoma of the peritoneum [10]. In our case the immunophenotipic profile was conclusive of MM: neoplastic cells were positive for calretinin, CK5 and D2-40 [11, 12]. These antibodies are known to be highly sensitive and specific immunohistochemical markers of MM and permit to exclude serous psammocarcinoma of the peritoneum. The behavior of serous psammocarcinoma is more closely similar to borderline serous tumor than to serous carcinoma. Further studies are necessary to establish if massive deposition of PBs may define a new variant of PMM with a favorable impact to the prognosis of usual PMM, as well as in serous psammocarcinoma of the peritoneum.
